# Spatial Pattern and Population Structure of *Artemisia ordosica* Shrub in a Desert Grassland under Enclosure, Northwest China

**DOI:** 10.3390/ijerph15050946

**Published:** 2018-05-09

**Authors:** Jiankang Liu, Kebin Zhang

**Affiliations:** 1 School of Soil and Water Conservation, Beijing Forestry University, Beijing 100083, China; liujiankang1989@126.com; 2 Key Laboratory of State Forestry Administration on Soil and Water Conservation, Beijing Forestry University, Beijing 100083, China

**Keywords:** enclosure, spatial pattern, population structure, desert grassland, recruitment

## Abstract

Enclosure is an effective practice for restoring and rehabilitating the degraded grassland ecosystem caused by overgrazing. Shrub species, which are dominant in most desert grasslands in arid and semiarid regions, have some beneficial ecological functions for grassland restoration. However, how the population structure and spatial pattern of the *Artemisia* *ordosica* shrub changes in a grassland ecosystem under enclosed practice is not well understood. This study, conducted in the Mu Us desert in northwest China, was designed to measure the *A. ordosica* population according to the chronosequence of enclosure (enclosure periods ranged from 5 years, 10 years, 15 years, and 25 years), contrasting this with an adjacent continuously grazed grassland. The results showed that the enclosed grasslands had a higher number of individuals of different age classes (seedling, adult, aging, and dead group) and greater population coverage, but shrubs had significant lower (*p* < 0.05) crown diameter and height in comparison with those in continuously grazed grassland. Further, enclosed grasslands had a significantly higher (*p* < 0.05) Shannon-Wiener index (H) and Evenness index (E), but a significantly lower (*p* < 0.05) Richness index (R) than continuously grazed grassland. The crown of *A. ordosica* showed a significant linear positive correlation with height in all plots across succession, indicating that it was feasible to analyze the age structure by crown. The crown-class distribution structure of the *A.* *ordosica* population approximated a Gaussian distribution model in all survey plots. Within the population, seedling and adult groups exhibited aggregated spatial distribution at small scales, while aging and dead *A. ordosica* groups showed random distribution at almost all scales in different plots. The seedling *A. ordosica* group showed a positive correlation with adults at small scales in all plots except in 10 years of enclosure. However, it showed independent correlation with aging and dead groups at almost all scales. In long-term enclosed plots, the mortality rate of the *A. ordosica* population increased, therefore assistance management practices, such as fertilization, mowing, interval grazing, and seasonal grazing, must be employed to maintain population stability after long-term enclosure. This study can improve understanding and clarify the effects of enclosures in the desert grasslands of northwest China.

## 1. Introduction

Grassland, which occupies approximately 37% of the earth’s terrestrial surface [1], is one of the world’s largest vegetation types. It is an important and essential natural ecosystem, which plays a key role in biodiversity conservation, socioeconomic development, greenhouse gas balance, food supply, and regional ecosystem stability [1,2,3]. However, due to climatic variation (global warming, droughts) [4,5,6] and human activities (plowing, deforestation, overcutting, and overgrazing) [7,8,9], grassland ecosystem degradation (characterized by the reduction of vegetation coverage, productivity, biodiversity and ecosystem services, and soil nutrient degradation) has become a major environmental issue all over the world in the recent decades [10,11,12,13,14]. Previous studies indicated that 49.3% of the world’s grasslands has been degraded to different degrees of severity (cf. 31.8% in China) [4,7]. Mu Us desert, as an important ecological barrier in northwest China, plays an important role in the national protection of wind erosion and sand storms [15]. However, due to increasing anthropogenic activities in this region, the grassland ecosystem has seriously deteriorated and become a crucial problem to be solved in recent decades [16,17]. 

Although a variety of practices, such as reseeding, plowing, fertilizing, establishing artificial grasslands, controlling grazing intensity, and rational management of the grassland, have been implemented to restore degraded grassland in Mu Us desert, enclosure has been widely considered as the most useful measure for low cost, simple implementation and quick effects [18,19,20]. Enclosure can increase plant biomass (above- and below-ground biomass) [21,22], improve plant diversity and soil physicochemical properties [19,23,24], promote ecosystem resilience [25] through livestock exclusion, and improve the livelihood of environmental conditions [26,27].

Vegetation restoration of degraded ecosystems is the joint action progress of plant–plant interactions [28,29], plant–soil feedback responses [30,31], and the facilitation of or interference between natural communities and environment [32,33]. The population structure and spatial pattern are the specific performances of comprehensive interactions among intra- and inter-specific interactions, species characteristics, interference, and environmental factors over the long term [34,35,36,37]. The population structure not only reflects the configuration and population dynamics of individuals, but also provides an important scientific basis for predicting the succession and development trend of populations [37,38]. The spatial pattern and correlation of species which represent the interaction between plants significantly affect the recruitment, growth, death, and resource utilization of species [36], and might depend on biological characteristics of plants and environmental heterogeneity [35,39]. Those two features can be used for dynamic analysis of vegetation [40].

Shrub species, which are dominant in most desert grasslands in arid and semiarid regions [41,42], are the crucial element in rehabilitating a degraded grassland ecosystem. They can provide shelter for other species [43,44] and significantly improve seedling survival for its “nursing” feature in arid and semiarid regions [45,46], eventually facilitating more complex vegetation restoration. *Artemisia ordosica*, a native subshrub species, is the main dominant species widely distributed in the Mu Us desert [15]. Therefore, the study on the *A. ordosica* shrub species is important for understanding the progress of vegetation restoration in this study area. Recent research on *A. ordosica* population dynamics mostly concentrated on natural recruitment [15,47], effects of habitat changes [15,48,49], spatial pattern [50,51], plant morphology and structural characteristics [52], soil moisture characteristics [53,54], and soil physicochemical properties [55]. However, the dynamics of the population structure and spatial pattern of the *A. ordosica* population in a restored desert grassland after removing livestock have not received enough attention. 

To better understand and clarify the effects of enclosure on the *A. ordosica* population in the southeast Mu Us desert, different periods of enclosure and one comparable, continuously grazed grassland were selected to analyze spatial pattern and population structure. The objectives of this study were to: (1) assess how population structure and spatial pattern of *A. ordosica* change after enclosure and (2) reveal the recruitment mechanism of the *A. ordosica* population across the succession time. The main findings of this study aim to provide the scientific basis and theoretical support for grassland restoration in Mu Us desert and to provide policy guidance for the restoration and management of degraded grasslands in other arid and semiarid regions around the world.

## 2. Materials and Methods

### 2.1. Study Site

The study was conducted in an artificially fenced area (37°50′ N, 107°23′ E) located in the Mu Us desert, northwest China. The elevation is approximately 1395 m. The local climate is classified as temperate continental, characterized by frequent drought, low precipitation, strong evaporation, short and hot summers, and long and cold winters. The annual precipitation mainly ranges from 250 mm to 350 mm. More than 80% of the precipitation occurs in the growing season of plants (from June to September). The mean annual evapotranspiration is 2024 mm. The annual mean air temperature is 8.1 °C, with the highest (16.2 °C) and lowest (−14.4 °C) monthly mean temperatures in July and January, respectively. The average frost-free period is about 165 days. The annual mean wind speed is 2.8 m/s, but bouts of wind over 6 m/s are common and dominated by west and northwest winds. The dominant species in the study area are some shrubs, semishrubs, or perennial herbs, such as *Artemisia ordosica*, *Heteropappus altaicus*, *Caragana Korshinskii*, *Artemisia scoparia*, and *Salix psammophila*. *A. ordosica* is the main focus of study area.

*A. ordosica* is a long-lived (about 10 years), deciduous, dwarf perennial shrub with linearly lobate leaves and is widely distributed in Mu Us desert, China [15,56,57]. It is easier to be grazed by livestock in the winter and spring seasons when forage grass is absent, although the palatability is poor during the growth season of plants. The flowering season mainly occurs around August and lasts approximately 50 days. The peak growing season is around July. *A. ordosica*, a low-growing (usually less than 100 cm) shrub species, has some paratactic branches near the ground [50,58]. Its root system is mainly distributed in the 30 cm of upper sand [47,48]. *A. ordosica* is generally recruited through seeds [59], which are tiny, light, and susceptible to wind-dispersion of more than several miles [60].

### 2.2. Study Design

Owing to excessive grazing by local Ning-Xia Tan sheep, local vegetation in the study area has been degraded seriously since late 20th century. Enclosure is the main practice implemented to restore the ecological system and the livelihood of the environment. We encircled the grassland with a chain link fence to remove herbivores such as local Ning-Xia Tan sheep, which are generally grazed by herdsman. However, there were still many small wild herbivores in enclosed grassland, such as rabbit and mice. The space-for-time method was used to monitor the effects of enclosure on the *A. ordosica* population. In this study, four successional ages, including enclosed 5 years (F5), 10 years (F10), 15 years (F15), and 25 years (F25), contrasting with adjacent continuously grazed grassland (CG), were selected for the plant survey (Table 1). Before enclosure establishment, the study area was grazed under similar intensity ensuring uniform natural conditions over the whole relatively flat study site. The five selected plots were contiguous. All plots were in the same continuous flat area and have the same slope, altitude, and soil type, insuring that their edaphic conditions were comparable before enclosure—although these conditions later changed after enclosure (Table 1). The vegetation coverage of the enclosed grassland was higher than that of continuously grazed grassland. All vegetation surveys were conducted in mid-August 2017, the peak growing season of plants.

**Figure 1:**
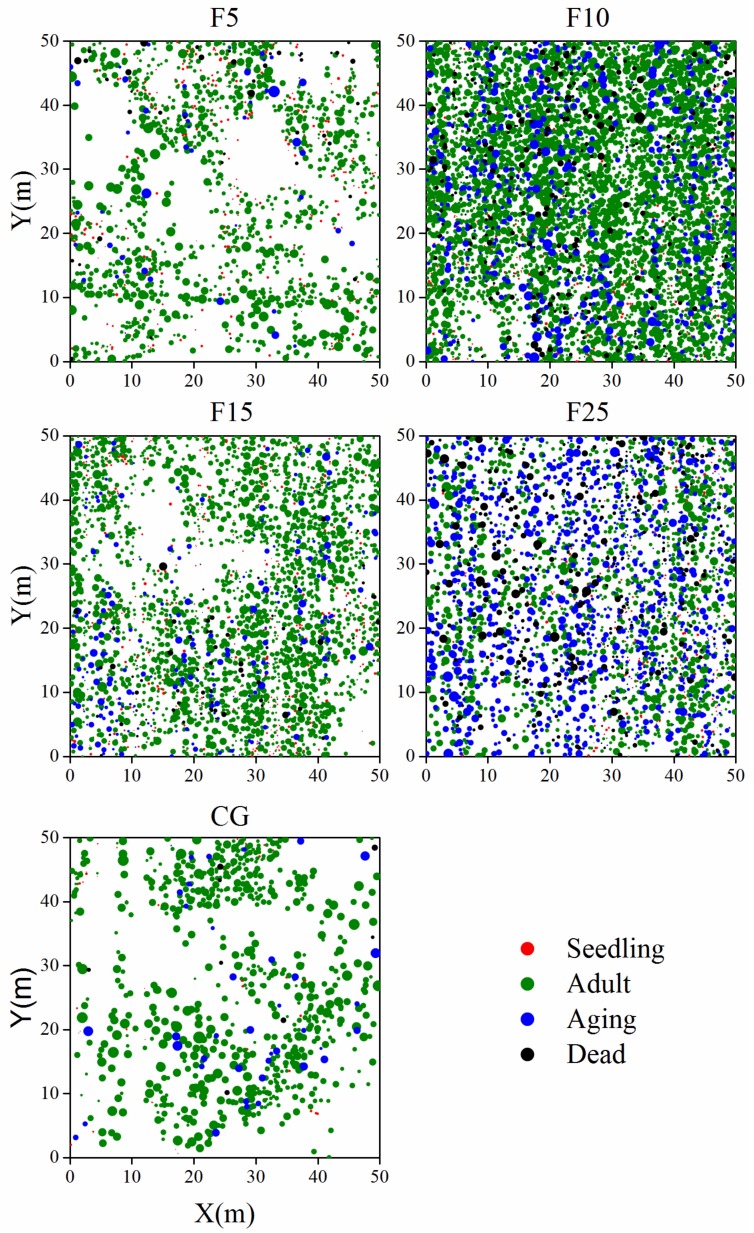
Maps of seeding, adult, aging, and dead *A. ordosica* groups in F5 (enclosed 5 years), F10 (enclosed 10 years), F15 (enclosed 15 years), F25 (enclosed 25 years), and CG (continuously grazed) plots. Symbol sizes represent the crown of individuals.

Data were collected on five 0.25 ha plots (50 × 50 m), each of which was established randomly for vegetation survey at F5, F10, F15, F25, and CG, respectively. *A. ordosica* was the dominant species in these five sample plots. Each sample plot was divided into 100 contiguous 5 × 5 m subplots. In each subplot, relative location coordinates, height (H), wide (WC) and narrow (NC) crown diameters, and the health status of each *A. ordosica* was recorded. The crown diameter of the shrub was calculated as WC/2 + NC/2 [61,62], like many other studies [15,49,63,64]. The height of the shrub was measured at the center of the crown where it is maximum [63,64]. All individuals, including seedling, adult, aging, and dead groups were crown-mapped (Figure 1). Further, in each selected plot, 10 random quadrats (1 × 1 m) were selected for an herb layer survey. In each quadrat, the density of each plant species was recorded.

### 2.3. Data Analysis

#### 2.3.1. Population Structure

*A. ordosica*, a subshrub species, does not produce notable annual rings [51]. Therefore, the morphological characteristics were used to distinguish age structures. In this study, crown classes were used to explain the age structures of *A. ordosica* population [65]. All *A. ordosica* individuals were classified into different classes (Class 1: from 0.1 cm to 10 cm; 10 cm were added gradually from Class 2 to 4 age groups (seedling group: average heights and crown were less than 30 cm; adult group: average heights and crown were more than 30 cm, and the dead branches proportion was less than two-thirds; aging group: the dead branches proportion was more than two-thirds; and dead group: individual which was lifeless) which were consistent with previous research [15,51,65,66]. The relationship between crown and height was fitted by a linear model. A Gaussian model was used for the curve fitting of the crown distribution structure to analyze the population dynamics.

#### 2.3.2. Spatial Pattern Analysis

The univariate spatial autocorrelation and intraspecific spatial correlation of different age groups were analyzed at multiple scales by the pair-correlation function *g*(r) instead of the commonly used Ripley’s K-function in this study. The g-function, which is related to the derivative of the K-function, is more sensitive to small-scale effects in comparison with K-function [67,68,69]. The formula of function *g*(r) is as follows [39,70]:

(1) $$g\left(r\right)=\frac{1}{2\pi t}\frac{A^{2}}{n^{2}}\overset{n}{\underset{i=1}{\sum }}\overset{n}{\underset{\begin{array}{c} i=1\\ j\neq 1 \end{array}}{\sum }}w_{ij}{}^{-1}k_{h}\left(t-\left|x_{i}-x_{j}\right|\right)$$

where *A* represents the plot area, *n* is the total plants number, and *w_ij_* means a weighting factor correcting for edge effects. *k_h_*, a kernel function, is used for applying maximum weight to point pairs within a distance *t*.

The g-function, based on point-to-point distances analysis, describes aggregating, random, or regularity spatial distribution within a given radius r by using a standardized density [71]. At a given distance *r*, *g*(r) > 1 indicates an aggregation trend, *g*(r) = 1 indicates complete spatial randomness (CSR), and *g*(r) < 1 indicates a regularity trend. To test significance departures of CSR, the 5th lowest and 199th highest values from the Monte Carlo simulations approach was used to generate approximately two-sided 95% simulation envelopes after calculating for each distance *r* of a point process.

#### 2.3.3. Species Diversity Index

The Shannon-Wiener (*H*), Richness (*R*), and Evenness indices (*E*) were used to illustrate species diversity [25]. These species diversity indices were calculated as:

Richness index (*R*):

*R* = S (2)

Shannon-Wiener index (*H*):

(3) $$H=-\overset{S}{\underset{i=1}{\sum }}\left(P_{i}lnP_{i}\right)$$

Evenness index (*E*):

*E = H/lnS* (4)

where *S* is the total species number found in each quadrat and *P_i_* is the density proportion of *i*th species.

All descriptive statistical parameters were carried out using EXCEL 2013. The significance test (*p* < 0.05) was calculated by SPSS 20.0 software (SPSS for Windows, Chicago, IL, USA). Spatial analysis was completed by Programita Febrero 2014. All figures were plotted by OriginPro 2015 (OriginLab Corporation, Northampton, MA, USA).

## 3. Results

### 3.1. Population Structure of A. ordosica Populations

The data showed that enclosure could significantly influence the individuals of different groups, coverage, crown, and height of the *A. ordosica* population (Table 2, Figure 1). Enclosed plots (F5, F10, F15, and F25) had more individuals of different age classes (seedling, adult, aging, and dead group), and greater population coverage, with significantly lower crown and height (*p* < 0.05) than the continuously grazed plot (CG). With the increase of enclosure time, the number of adult and total *A. ordosica* increased initially, and then decreased. Both parameters were highest in the F10 plot (3523 adult individuals, 4643 total individuals). In contrast, the proportion of aging (34.1%) and dead (13.4%) groups in the F10 plot, and the proportion of the seedling group (19.1%) in the F5 plot was higher than the other four plots. The proportion of adults was minimal in the F25 plot (45.0%), while maximal in the CG plot (87.0%). The F5 plot had the lowest population coverage (19.7%) and height (0.289 ± 0.102) of the four enclosed plots. The crown and height of the F10 plot were significantly higher (*p* < 0.05) than the other three enclosed plots (F5, F15, and F25). The F15 plot had minimum crown (0.556 ± 0.221) (Table 2, Figure 1).

**Figure 2:**
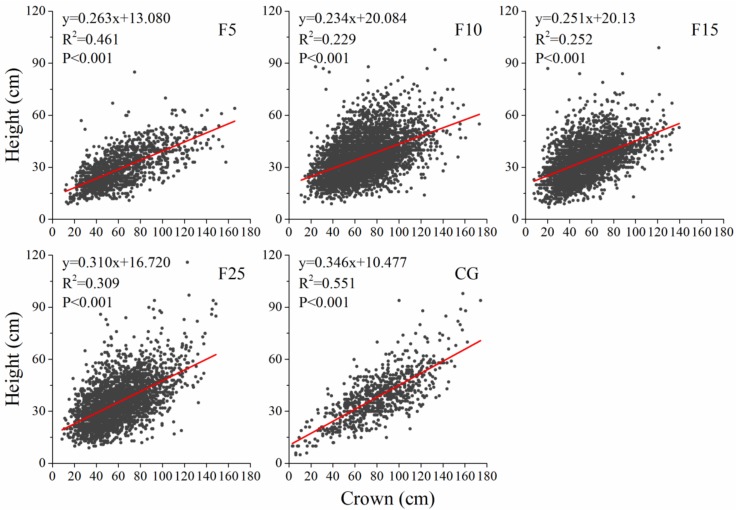
The crown and height allometric model of *A. ordosica* population in F5 (enclosed 5 years), F10 (enclosed 10 years), F15 (enclosed 15 years), F25 (enclosed 25 years), and CG (continuously grazed) plots.

There was a significant linear positive correlation (*p* < 0.001) between crown and height of the *A. ordosica* population in all plots. The regression slope (0.346) of linear equation in continuously grazed plot was higher than that in the enclosure plots (F5: 0.263, F10: 0.234, F15: 0.251, and F25: 0.310). The slope of the linear equation initially declined in the first 10 years after enclosure and subsequently slowly increased (Figure 2).

**Figure 3:**
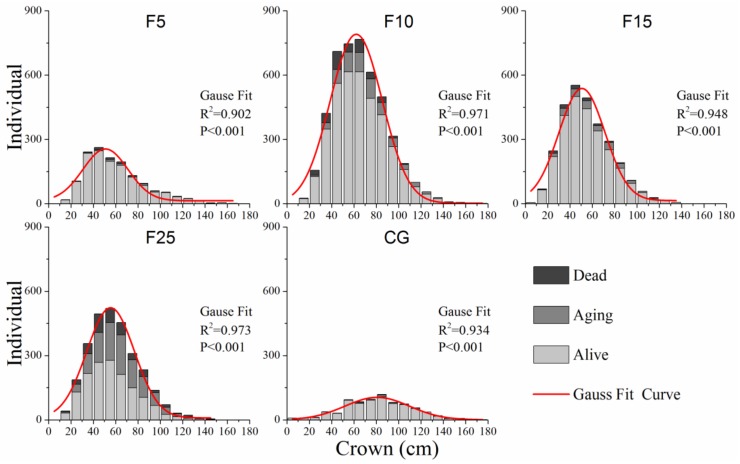
Crown distribution structure of alive, aging, and dead *A. ordosica* in F5 (enclosed 5 years), F10 (enclosed 10 years), F15 (enclosed 15 years), F25 (enclosed 25 years), and CG (continuously grazed).

According to the polynomial fitting results (Figure 3), the crown distribution structure of the *A. ordosica* population approximated a Gaussian distribution model in all survey plots (*R*^2^ > 0.9, *p* < 0.001). Probably due to differences in the site environmental conditions after long-term enclosure, *A. ordosica* grew smaller in the F15 and F25 plots (up to crown = 140 cm) than in the F5 and F10 plots. The main distribution of crown is within 30–120 cm. Seedling and medium-sized (crown within 40–100 cm) *A. ordosica* thrived in the F5, F10, and F15 plots, which suggests a stable, renewable population. The dead and aging individuals of medium-sized *A. ordosica* were larger than seedlings and big-sized adults (crown more than 100 cm). At the F25 plot, the alive rate of individuals was lower than other survey plots (Figure 3).

### 3.2. Spatial Autocorrelation of A. ordosica Populations

**Figure 4:**
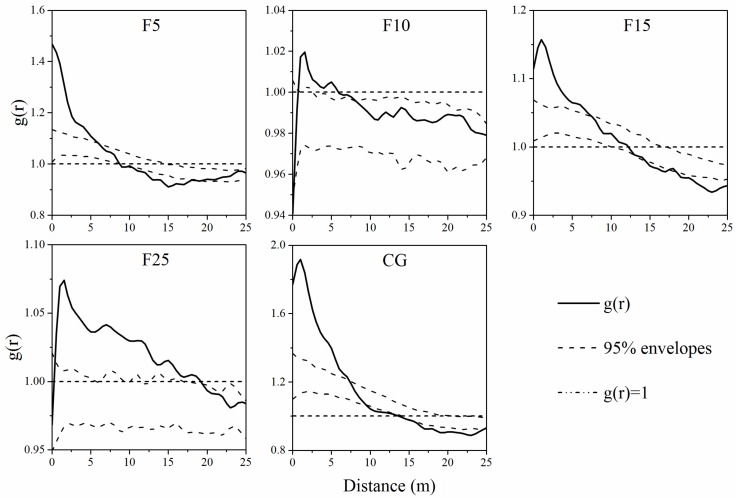
Spatial autocorrelation of entire *A. ordosica* population on F5, F10, F15, F25 and CG plots. F5 (enclosed 5 years), F10 (enclosed 10 years), F15 (enclosed 15 years), F25 (enclosed 25 years), and CG (continuously grazed).

**Figure 5:**
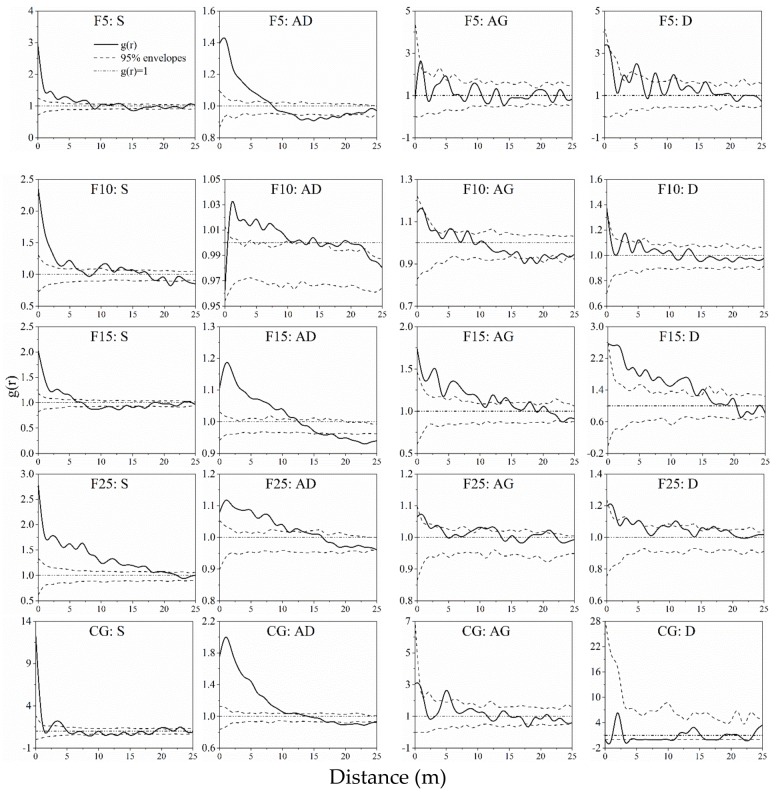
Univariate spatial autocorrelation of four age classes of the *A. ordosica* population on the F5, F10, F15, F25, and CG plots. S: seedling group, AD: adult group, AG: aging group, and D: dead group. F5 (enclosed 5 years), F10 (enclosed 10 years), F15 (enclosed 15 years), F25 (enclosed 25 years), and CG (continuously grazed).

**Figure 6:**
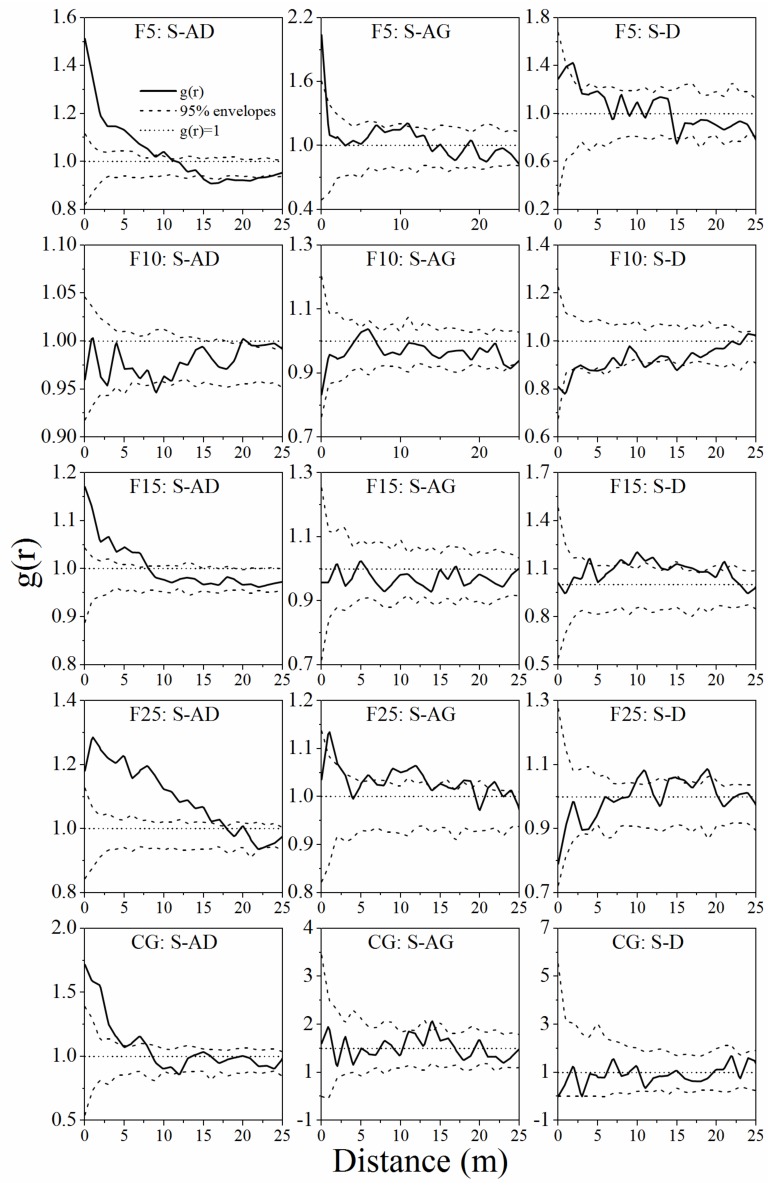
Intraspecific spatial correlations among different age classes of the *A. ordosica* population on F5, F10, F15, F25, and CG sites. S: seedling group, AD: adult group, AG: aging group, D: dead group. F5 (enclosed 5 years), F10 (enclosed 10 years), F15 (enclosed 15 years), F25 (enclosed 25 years), and CG (continuously grazed).

The univariate spatial autocorrelation and intraspecific spatial correlation of the entire population, S (seedling groups), AD (adult groups), AG (aging groups), and D (dead groups) were unlikely in five survey plots (Figure 4, Figure 5 and Figure 6). Entire population, seedling, and adult groups exhibited aggregated distribution at small scales, especially at scales of 0–5 m (Figure 4 and Figure 5). The aging and dead *A. ordosica* groups showed random distribution at almost all scales in the F5, F10, F25, and CG plots, while those two groups showed aggregated distribution within 0–11 m and 12–15 m in the F15 plot (Figure 5). The aggregation degree of *A. ordosica* adult groups was greater than that of entire and seedling populations in the F10, F15, and CG plots, but was less in the F5 and F25 plots (Figure 4 and Figure 5). Further, the aggregation degree of *A. ordosica* seedling groups declined with the distances increasing in all plots (Figure 5). The seedlings of *A. ordosica* showed positive correlations with adults at small scales in all plots except for F10, especially at scales of 0–5 m, although they showed independent correlations at almost all scales with aging and dead *A. ordosica* (Figure 6).

### 3.3. Species Diversity of Community

**Figure 7:**
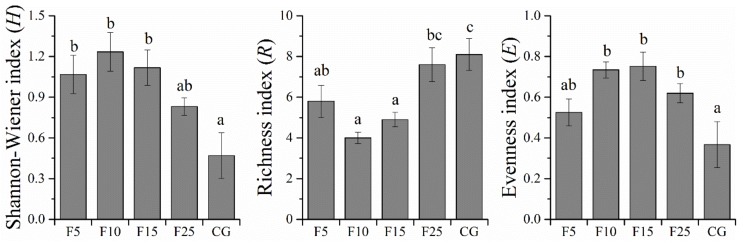
The Shannon-Wiener index (H), Richness index (R), and Evenness index (E) of communities in F5 (enclosed 5 years), F10 (enclosed 10 years), F15 (enclosed 15 years), F25 (enclosed 25 years), and CG (continuously grazed) plots.

In this study, enclosure grasslands (F5, F10, F15, and F25) had significantly higher (*p* < 0.05) Shannon-Wiener (H) and Evenness (E) indices but had a significantly lower (*p* < 0.05) Richness index (R) than continuously grazed grassland (CG). Further, with the increase of enclosure time, H and E indices were all increased initially, and then decreased, which were highest in the F10 plot and the F15 plot, respectively. However, the R index decreased initially and then increased with the increase of enclosure time, which was lowest in the F10 plot (Figure 7).

## 4. Discussion

### 4.1. Population Structure of A. ordosica Population

Population structure is the synthetic action of biological characteristics, environmental factors, and intraspecific interactions [40,72] which can be used to reflect the dynamics and development trends of a population [37]. Some essential characteristics of plants, such as density, height, crown, and diameter at breast height (DBH), can reflect the growth status and ecological functions of plants and affect the ecological process of a population, community, or even an ecosystem [73,74,75]. With the gradual restoration of an ecological environment after enclosure, the vegetation community gradually recovered [23,24,26,27], characterized by a significant increase (*p* < 0.05) of Shannon-Wiener and Evenness indices and rapidly growing development of the *A. ordosica* population (Table 2, Figure 1 and Figure 7). In the early stage of restoring grassland, natural conditions are favorable for the recruitment of *A. ordosica* because of adequate resource supply and lack of competition from other species [42,71]. However, the massive reproduction of *A. ordosica* restricted the growing development of plant species with weak competitiveness. As shown in this study, for individuals and coverage of the *A. ordosica* population, Shannon-Wiener and Evenness indices increased quickly, but the Richness index greatly reduced in the first 10 years after enclosure. At this time, a great deal of seeds germinated and grew into seedlings or adults, which caused a high reduction of shrubs’ height and crown (Table 2). However, elevated plant density and size lead to some individuals dying or withering because of a lack of ability to face intense intraspecific competition for light, water, and nutrients [76,77,78]. Further, the appearance and development of soil crusts, which is water-absorbing and hinders water infiltration, would prevent seeds from germinating [79,80,81]. Therefore, for the individual seedlings and adults, the population coverage, height, crown diameter, Shannon-Wiener index, and Evenness index decreased, while the proportion of aging and dead individuals of different-sized *A. ordosica* increased after being enclosed for 25 years (Table 2, Figure 3 and Figure 7). This reduction of dominant species reduced competitive pressure on other species [25], so the Richness index increased after being enclosed for 25 years (Figure 7).

The allometric model of plant characteristics can reflect the plastic responses of plants to resource availability and the natural selection or ecological sorting of species in the competitive environment of local resources [82,83]. This is the long-term response of the interaction between plants and environment [84]. As shown in Figure 2, the regression model indicated how plant height changed with crown. The data showed that the linear equation slope in enclosure plots was lower than the continuously grazed plot (Figure 2), which was probably caused by the removal of livestock activities such as ingestion and trampling [85]. The significant positive linear correlation between height and crown of *A. ordosica* indicated that it was feasible to analyze age structure by crown distribution.

As a dominant shrub species in Mu Us desert, *A. ordosica* adapts well to the local environment [15]. Our study showed that the crown distribution structure of the *A. ordosica* population approximated a Gaussian distribution model (Figure 3). In addition, the abundance of seedling and medium-sized *A. ordosica* demonstrated a stable crown-diameter distribution structure in enclosed grassland (Figure 3) [40]. The exclusion of livestock had little effect on crown-diameter distribution structure across the succession time, but it could influence the recruitment and mortality of the population (Figure 3).

### 4.2. Spatial Pattern of A. ordosica Population

The spatial pattern of plants is the performance of comprehensive interactions between plants and environmental factors over a long term, which can be used to reveal the succession law of vegetation [35,36,37,40,86,87]. The spatial-patterns structure analysis of different growth stages can obtain some essential information of a population under natural or human disturbance, such as recruitment, wilting or mortality of individuals, and intraspecific competition [51,70,88,89]. In this study, different age *A. ordosica* groups showed unpredictable spatial patterns in enclosed and grazing grasslands. The entire *A. ordosica* population, seedling, and adult groups showed significantly aggregated trends at small scales but random or even a uniform trend at large scales in all survey plots (Figure 4 and Figure 5), which was consistent with previous studies in this area [15,51]. This is mainly determined by plant–plant interactions [90] and patches resources [91]. Moreover, because of the limited resources and spaces for reproduction and expansion, aggregated distribution at small patches is beneficial for individuals, especially for seedlings, to resist adverse nature conditions [15,42].

The spatial distribution pattern of plants is closely related to human or animal disturbance [88,89,92], such as grazing, which is widely considered to be one of the most direct and essential factors for vegetation succession [25,93,94]. Removal of livelihood usually led to the reduction of environmental spatial heterogeneity [95]. Therefore, the aggregation degree of the *A. ordosica* population in the F10 plot was lower than that in the F5 and CG plots. Further, the random mortality pattern of plants [96] results in a random distribution of aging and dead groups (Figure 5). With the ecological environment gradually improved after the grassland was enclosed, the *A. ordosica* population rapidly developed (Table 2) due to the reduction of environmental stress (i.e., sand burial, wind erosion, water erosion) [26,27,51]. The elevated shrub density caused nonrandom intraspecific competition [97,98], characterized by an aggregation of dead and aging groups in the F15 plot (Figure 5). Therefore, with the continuation of enclosure, the surviving *A. ordosica* showed an even more aggregated spatial autocorrelation pattern in the F25 plot (Figure 5 and Figure 6).

It is likely that adult groups were relatively important for *A. ordosica* population recruitment in this study (Figure 6). The seed of *A. ordosica* is tiny and light and has some special structures to allow airborne dispersal [60]. The seeds are more likely to be caught by adult shrubs, and then generate and survive under or nearby the canopy because of the amelioration of the microenvironment [99,100,101]. Moreover, large shrub patches can provide a shelter for seedlings under canopy and protect the seedlings from water–wind erosion and sand burial [102,103,104]. However, when shrubs are wilted or dead, the nursing effects will diminish [105]. Spatial pattern analysis could well support this theory. From the results of this study, *A. ordosica* seedlings had an aggregated distribution within 0–5 m and had a significant positive correlation with adults at small scales (0–5 m) at the beginning of grassland enclosure (Figure 5 and Figure 6). The seedling group showed independent correlation with aging and dead groups (Figure 6). Soil nutrients were gradually enriched under shrub canopy after enclosure because they intercepted atmospheric dust and litter decomposition [106]. Therefore, the soil crust cover and thickness under shrub canopy are larger than in open spaces nearby [107], which limit the germination of seeds [100]. So, after enclosure for 10 years, the spatial correlation between seedlings and adults became independent (Figure 6). At this time, limited resources result in density-dependent self-thinning, although spatial heterogeneity (i.e., microtopography, soil, and water) causes nonrandom mortality of plants [40,71]. Therefore, some individuals gradually died because of increasing competition, leading to a reduction of population density (Table 2, Figure 3 and Figure 4). Finally, after 15 years of enclosure, the seedlings were positively associated with adults at small scales (spatially) and changed to an independent correlation at large scales (Figure 6).

## 5. Conclusions

Enclosure is an effective practice to recover the *A. ordosica* population in Mu Us desert. It can increase Shannon-Wiener and Evenness indices while decreasing the Richness index. Moderate enclosure had positive effects on the *A. ordosica* population in degraded grazing grassland, including an increase in the seedling ratio, population individuals, population coverage, and reduction in the height–crown ratio. However, the aging and death rate of the population increased after a long period of enclosure. The entire population, seedling, and adult *A. ordosica* groups showed an aggregated distribution at small scales, although aging and dead groups were randomly distributed at almost all scales in different plots. The seedling *A. ordosica* group showed a significant positive correlation with adults, while independent correlation with aging and dead groups. Enclosure is an important factor affecting the spatial autocorrelation and intraspecific spatial correlation of alive *A. ordosica*. With the continuation of enclosure, the surviving *A. ordosica* showed a more aggregated spatial autocorrelation pattern. Further, the spatial correlation between seedlings and adults changed from positive at small scales in the F5 plot to independent at all scales in the F10 plot and eventually became more positive at small scales. Those results indicated that continuous long-term enclosure might cause re-degradation of the *A. ordosica* population. Therefore, other artificial measures, such as fertilization, mowing, interval grazing, and seasonal grazing, should be implemented to maintain the stability and sustainability of shrub-dominated grasslands after restoration.

## Figures and Tables

**Table 1:** Basic characteristics information in different plots. F5 (enclosed 5 years), F10 (enclosed 10 years), F15 (enclosed 15 years), F25 (enclosed 25 years), and CG (continuously grazed).

Plot Number	Coverage (%)	Slope	Altitude	Soil Type	Geographic Coordinates	
					Latitude (N)	Longitude (E)
F5	37	1–3°	1393 m	Sierozem	37°50′54.4”	107°23′12.7”
F10	75	1–2°	1393 m	Sierozem	37°50′49.9”	107°23′49.0”
F15	40	1–3°	1394 m	Sierozem	37°50′46.3″	107°23′48.3″
F26	48	1–3°	1396 m	Sierozem	37°50′46.1″	107°24′07.8″
CG	25	2–4°	1395 m	Sierozem	37°50′40.5″	107°24′31.3″

**Table 2:** Category and basic parameters of *A. ordosica* population in five plots, F5 (enclosed 5 years), F10 (enclosed 10 years), F15 (enclosed 15 years), F25 (enclosed 25 years), and CG (continuously grazed).

Plot Number	Population Individuals of Different Groups					Population Coverage (%)	Crown (m)	Height (m)
	Seedling (%)	Adult (%)	Aging (%)	Dead (%)	Total			
F5	279 (19.1)	1089 (74.7)	46 (3.2)	44 (3.0)	1458	19.7	0.601 ± 0.262 b	0.289 ± 0.102 a
F10	257 (5.5)	3523 (75.9)	536 (11.5)	327 (7.0)	4643	71.7	0.661 ± 0.236 c	0.355 ± 0.115 c
F15	387 (13.3)	2201 (75.9)	229 (7.9)	83 (2.9)	2900	32.6	0.556 ± 0.221 a	0.341 ± 0.111 b
F25	216 (7.5)	1294 (45.0)	980 (34.1)	385 (13.4)	2875	36.7	0.596 ± 0.226 b	0.352 ± 0.126 c
CG	53 (6.8)	678 (87.0)	40 (5.1)	8 (1.0)	779	18.3	0.814 ± 0.294 d	0.387 ± 0.138 d

Data are presented as the mean ±SD. Different small letters in same column indicated significant differences (*p* < 0.05).
